# Unravelling the Dynamic Physiological and Metabolome Responses of Wheat (*Triticum aestivum* L.) to Saline–Alkaline Stress at the Seedling Stage

**DOI:** 10.3390/metabo15070430

**Published:** 2025-06-23

**Authors:** Wei Ren, Li Chen

**Affiliations:** 1 State Key Laboratory of Ecological Safety and Sustainable Development in Arid Lands, Xinjiang Institute of Ecology and Geography, Chinese Academy of Sciences, Urumqi 830011, China; chenli@ms.xjb.ac.cn; 2 Fukang Station of Desert Ecology, Chinese Academy of Sciences, Fukang 831505, China

**Keywords:** wheat, salt–alkali soil, phytohormone, differential metabolite, metabolic pathways, LC-MS

## Abstract

Background/Objectives: Understanding metabolome adjustment under saline–alkaline conditions is crucial for enhancing crop tolerance capacity and ensuring food security. Although soil salinization impairs wheat seedlings’ growth, metabolome plasticity under saline–alkaline stress remains poorly understood. Here, we delved into dynamic physiological and metabolome shifts in wheat seedlings grown on SAS (saline–alkaline soil) on the 7th and 15th days post-germination (DPG). Methods: A self-developed and cultivated high-generation salt–alkali wheat variety (011) was grown on SAS and control soil, followed by comparative physiological, biochemical, and metabolomics analyses of seedlings. Results: The seedlings’ saline–alkaline stress responses were developmentally regulated with reduced growth, increasing accumulation of proline and soluble sugars, and differential antioxidant response. LC-MS-based global metabolomics analysis revealed significant metabolite profile differences, with 367 and 485 differential metabolites identified on the 7th and 15th DPG, respectively, between control and treatment. Upregulation of saccharides, flavonoids, organic acids (citrate cycle-related), phenolic acids, amino acids and derivatives, phytohormones, and sphingolipid metabolism was essential for seedlings’ growth on SAS. The key induced metabolites in seedlings grown on SAS include saccharic acid, trehalose, sucrose, glucose, L-citramalic acid, phellodendroside, scutellarin, anthranilate-1-*O*-sophoroside, lavandulifolioside, N-methyl-L-glutamate, etc. Up-regulated phytohormones include abscisic acid (3.8-fold, 7th DPG and 3.18-fold, 15th DPG), jasmonic acid (1.93-fold, 15th DPG), and jasmonoyl isoleucine (2.03-fold, 15th DPG). Conclusions: Our findings highlight the importance of ABA and jasmonic acid in regulating salt–alkali tolerance in wheat seedlings. Moreover, this study depicts key pathways involved in salt–alkali tolerance in wheat seedlings and unveils key DMs, offering resources for boosting wheat production on SAS.

## 1. Introduction

Improving and sustaining crop production is a major challenge in agriculture in the current context of climate change. Indeed, climate change is exacerbating soil salinization, which is one of the major threats to environmental sustainability and global food security [1]. Soil salinization is worse in arid regions, where it causes food crises due to the shortage of cultivated land resources, accelerated land degradation, and declined crop productivity [1,2]. Salinization of agricultural soils causes saline–alkaline stress, which severely affects plant growth and crop yields [3]. About 8.3 × 10^8^ Da of land is impacted by natural salinization worldwide [4]. In China, Xinjiang, located in the inland arid desert regions, includes 3.02 × 10^6^ Da of saline–alkali land, accounting for 37.72% of the total cultivated land [4,5,6]. The annual rise of saline–alkali land in this area is estimated at 0.26%, representing a major limit to agricultural development and food productivity [5]. The world’s most consumed and cultivated grains include rice and wheat, which have the potential for food and nutritional sustainability [7,8]. Particularly, wheat is a vital grain crop in Xinjiang, and it holds significant potential in the agricultural industry structure [6,9]. Therefore, explaining the wheat plant’s saline–alkaline tolerance mechanisms will help elaborate efficient strategies to promote its production on SAS.

Saline–alkaline stress harms plant growth and production than salinity stress alone, as it simultaneously induces ionic, oxidative, osmotic, and high pH stresses [3,10]. The synergistic effects of these stresses on plants include disruption of ion homeostasis, reduced nutrient uptake of mineral nutrition, increased cytoplasmic Na^+^ content, hampered proton pump, altered chloroplast structure, disrupted photosynthesis and energy metabolism, damaged cell structure, etc. [3,10,11]. Plants address saline–alkaline stress by activating the osmotic-regulation pathway and the energy metabolism pathway, resulting in metabolic adjustment related to ion transport, photosynthesis, phytohormone synthesis, antioxidant response, solute accumulation, and osmotic adjustment [3,10,12]. A low Na^+^/K^+^ ratio in plant cells is crucial to maintain ion homeostasis and prevent cellular damage and nutrient deficiency under saline–alkaline stress [3,10,13]. Plants also utilize diverse signaling components and sensors to alleviate salt stress promptly, most of which involve Ca^2+^ sensing and signaling [14,15]. ABA and GIPC (glycosyl inositol phosphorylceramide) sphingolipids promote Ca^2+^ signaling, leading to enhanced saline–alkaline tolerance [3].

In wheat, most previous studies concentrated on salinity-responsive mechanisms, and very little is known regarding saline–alkaline stress tolerance. Saline–alkaline stress impairs wheat seed germination and seedlings’ growth [16]. Root system and osmolytes, proline and soluble sugars, are vital for saline–alkaline tolerance in wheat [13,16]. Mourad et al. have unlocked the understanding of the genetic control of saline–alkaline tolerance in wheat and identified seven and 20 regulatory gene models at the seedling and mature growth stages, respectively [17]. Saline–alkaline stress regulation involves the interplay between genes and metabolites [18,19]. However, metabolome adjustments under saline–alkaline stress in wheat remain elusive. Hence, revealing metabolomic changes in response to saline–alkaline stress and identifying tolerance-related metabolites will provide hints for dissecting the molecular regulatory networks and resources to improve wheat tolerance capacity.

Here, we employed a self-developed saline–alkaline-tolerant wheat variety to investigate the dynamic physiological and metabolic changes in wheat seedlings grown on SAS compared to those grown on a control soil. We evaluated the germination rate and growth-related traits. Principally, we assessed physiological traits and examined metabolome patterns on the 7th and 15th DPG. Our objective was to gain more insights into the mechanisms underlying wheat tolerance to saline–alkaline stress at the seedling stage. Our findings will help expand wheat production on SAS.

## 2. Materials and Methods

### 2.1. Plant Materials and Plant Growth Conditions

A self-developed and cultivated high-generation salt–alkali wheat variety (011) was evaluated in this study. Fifteen seeds were sown in pots on SAS (salt–alkali treatment) and control soil. The SAS was taken from farmland located about ten kilometers north of Fukang County, a county-level city in the Changji Hui Autonomous Prefecture of Xinjiang Uygur Autonomous Region (newly cultivated for three years). No fertilizer was used. The physicochemical properties of the soils are presented in Table 1. Each treatment was repeated six times and maintained in a growth chamber at 30 °C/26 °C (day/night) under 16 h/8 h long-day conditions (light/darkness). Wheat seedlings grown on SAS were labelled “D”, while those on control soil were coded “A”. The germination rate (GR = seeds germinated/total seeds × 100) was assessed after six days. Seedlings’ growth characteristics (plant height and fresh weight) were evaluated on the 7th and 15th DPG (day post-germination). Seedling leaf samples were also collected in triplicate on the 7th and 15th DPG, respectively, to evaluate physiological traits and metabolite profiling. Samples were frozen in liquid nitrogen and stored at −80 °C until analysis.

### 2.2. Evaluation of Physiological Parameters

The investigated traits include proline content, total sugars content, malondialdehyde content, and the activity of SOD (superoxide dismutase) and CAT (catalase). All these traits were evaluated using relevant detection kits (Norminkoda Biotechnology Co., Ltd., Wuhan, China, http://www.bionmkd.com), following the manufacturer’s instructions.

### 2.3. Extraction of Samples for Metabolomics Analysis

Freeze-dried leaf samples were ground (MM 400, Retsch, Haan, Germany, 30 Hz for 1.5 min), and 50 mg of the sample powder was extracted with 1200 μL of 70% methanolic aqueous solution (pre-cooled at −20 °C). Vortexed six times, once every 30 min for 30 s. After centrifugation (12,000 rpm, 10 min), we aspirated the supernatant and filtered it using a microporous membrane (0.22 μm). Extracts were stored in the injection vial for UPLC-MS/MS analysis.

### 2.4. UPLC and ESI-Q TRAP-MS/MS Conditions

The metabolite profiling was performed at Norminkoda Biotechnology Co., Ltd., Wuhan, China, as per previously reported methods [20,21,22]. Briefly, we analyzed all extracts using a UPLC-ESI-MS/MS (UPLC, ExionLC™ AD, Shanghai, China) and a Tandem MS (mass spectrometry) system. The conditions were the following: UPLC: column, Agilent SB-C18 (Santa Clara, CA, USA, 1.8 µm, 2.1 mm × 100 mm); mobile phase was composed of solvent A (pure water with 0.1% formic acid) and solvent B (mixed acetonitrile and 0.1% formic acid). The measurement gradient program was:

- Starting conditions: 95% A, 5% B; within 9 min, a linear gradient to 5% A, 95% B; and a composition of 5% A, 95% B was kept for 1 min;
- Then, a composition of 95% A and 5.0% B was adjusted within 1.1 min and kept for 2.9 min;
- The flow velocity was set as 0.35 mL/min;
- The column oven was set to 40 °C;
- The injection volume was 2 μL;
- The effluent was alternatively connected to an ESI-triple quadrupole-linear ion trap (QTRAP)-MS.

The Electrospray Ionization (ESI) source system was set to source temperature 500 °C; ion spray voltage (IS) 5500 V (positive ion mode)/−4500 V (negative ion mode). The ion source gas I (GSI), gas II (GSII), and curtain gas (CUR) were maintained at 50, 60, and 25 psi, respectively. The CAD (collision-activated dissociation) was high. QQQ scans were acquired as MRM, with the collision gas (nitrogen) set to medium. Declustering potential (DP) and CE (collision energy) for individual MRM transitions were achieved via further DP and CE optimization. We monitored a specific set of MRM transitions for each period according to the eluted metabolites within that period.

### 2.5. Statistical Analysis

Metabolites with more than 20% missing values were excluded. A three-step process was then applied to remove redundant peaks, and the signal intensities were log-transformed prior to multivariate analyses in R (vs 4.3.0). The packages pheatmap and MetaboAnalystR were used for HCA (hierarchical clustering analysis) and OPLS-DA (orthogonal partial least squares discriminant analysis). The package prcomp was used for PCA (principal component analysis). DMs (differential metabolites) were screened out by the ggplot2 program (R software) at thresholds of *p*-value < 0.05, |Log2FC| > 1, and VIP ≥ 1. Induced pathways were unveiled by KEGG analysis (http://www.kegg.jp/kegg/pathway.html, accessed on 13 March 2025). Data were analyzed and plotted using Microsoft Excel and GraphPad Prism 9. Statistical significances (*p* < 0.05) were detected through ANOVA (analysis of variance), with post hoc Duncan test. TBtools (v2.121) was used to generate Venn diagrams and heat maps [23].

## 3. Results

### 3.1. Effect of Salt Stress on Wheat Seed Germination and Growth

**Figure 1:**
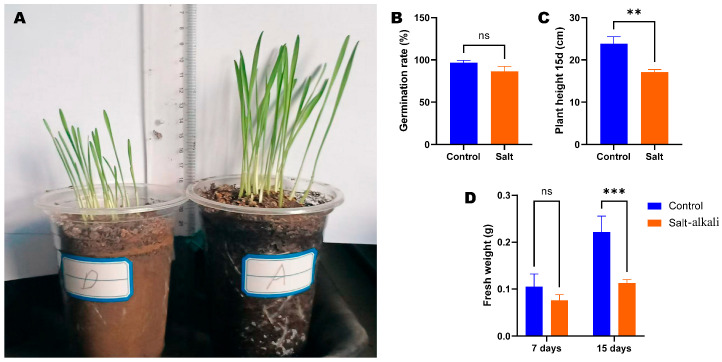
Impacts of salt–alkali stress on wheat seed germination and seedling growth. (**A**) Morphologies of wheat seedlings grown on saline–alkaline soil (D) and control soil (A). (**B**) Germination rates. (**C**) Plant height on the 15th day post-germination (DPG). (**D**) Fresh weight on the 7th and 15th DPG. Each bar represents mean ± standard deviation of three replications. Six replications were applied for the germination rate. Statistical significance was determined by ANOVA with a post hoc Duncan test. ** and *** indicate statistical differences at *p* ˂ 0.01 and 0.001, respectively. ns, no significant difference.

To reveal the effects of saline–alkaline stress on wheat plant development, we assessed seed germination and seedlings’ growth on the 7th and 15th DPG under control soil (A) and SAS (D) (Figure 1). The seed germination rate in SAS (87%) was slightly lower than under control conditions (97%), but the difference was not significant (Figure 1B). The seedling height and fresh weight on the 15th DPG under SAS were significantly lower compared to under control conditions (Figure 1A,C,D). For instance, the fresh weight of seedlings increased by 52.46% from the 7th to 15th DPG (Figure 1D). Unlike under SAS, it increased only by 32.5% (Figure 1D).

### 3.2. Dynamic Physiological Responses of Wheat Seedlings to Salt Stress

**Figure 2:**
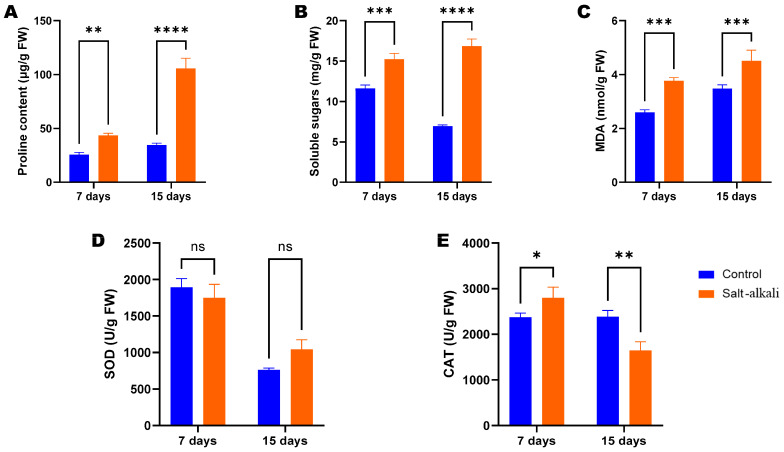
Impacts of salt–alkali stress on wheat seedling physiological traits on the 7th and 15th day post-germination. (**A**) Proline content. (**B**) Soluble sugar content. (**C**) Malondialdehyde content. (**D**) SOD activity. (**E**) CAT activity. Each bar represents mean ± standard deviation of three replications. Statistical significance was determined by ANOVA with a post hoc Duncan test. *, **, ***, and **** indicate statistical differences at *p* ˂ 0.05, 0.01, 0.001, and 0.0001, respectively. ns, no significant difference.

Improved physiological performance is crucial for the abiotic stress tolerance of plants. To tackle the dynamic physiological changes in wheat seedlings under SAS, we evaluated the content of osmoprotectants (proline and soluble sugars), malondialdehyde (MDA) and the activity of two antioxidant enzymes (SOD, superoxide dismutase and CAT, catalase) on the 7th and 15th DPG (Figure 2). The MDA, proline, and total sugar contents of wheat seedlings grown in SAS on the 7th and 15th DPG were significantly higher than for those grown on normal soil (Figure 2A–C). The activity of SOD in wheat seedlings grown in SAS on the 15th DPG was slightly higher than in those grown on normal soil (Figure 2D). The CAT activity in wheat seedlings grown in SAS on the 7th was significantly higher than in controls (Figure 2E). However, it showed the opposite trend on the 15th DPG (Figure 2D).

### 3.3. Metabolome Plasticity of Wheat Seedlings Under Salt Stress

**Figure 3:**
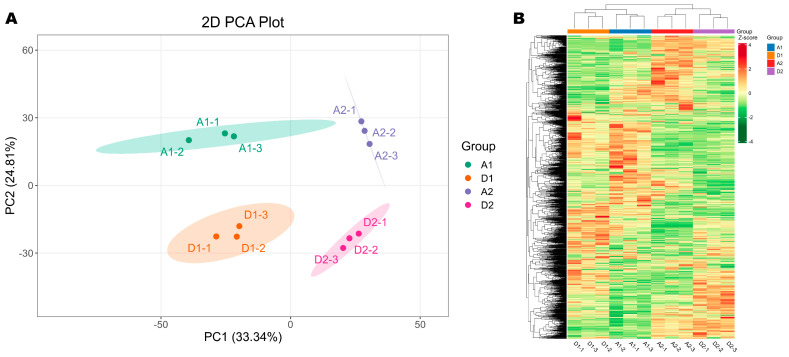
Impacts of salt–alkali stress on wheat seedling metabolite profile. (**A**) Principal component analysis. (**B**) Hierarchical clustering analysis. A1 and A2 indicate seedlings grown on the control soil on the 7th and 15th day post-germination, respectively. D1 and D2 indicate seedlings grown on the salt–alkali soil on the 7th and 15th day post-germination, respectively. Then, 1-1, 1-2, and 1-3 represent replications for A1 and D1. 2-1, 2-2, and 2-3 represent replications for A2 and D2.

To delve into the dynamic metabolome responses of wheat seedlings under salt–alkali stress conditions, we performed comparative metabolite profiling. The correlations among QC samples were equal or close to 1, indicating the reproducibility of the experiment (Figure S1). We identified a total of 2296 metabolites, including alkaloids (12.77%), amino acids and derivatives (12.24%), flavonoids (18.39%), lipids (11.42%), phenolic acids (10.07%), organic acids (4.58%), terpenoids (7.63%), etc. (Table S1, Figure S2). To explore the similarity of the metabolite profiles of seedlings grown in SAS and normal soil on the 7th and 15th DPG, we carried out HCA and PCA analyses. As shown in Figure 3A,B, the results indicate a clear difference in the metabolite profiles of the four groups. Seedlings grown on SAS and normal soil showed different metabolome patterns on the 7th and 15th DPG, which were clearly separated by the first two principal components (PC1, 33.34% and PC2, 24.81%) (Figure 3A,B). These results show a dynamic metabolome adjustment in wheat seedlings in response to prolonged salt–alkali stress.

### 3.4. Differential Metabolites (DMs) and Key Responsive Metabolic Pathways

**Figure 4:**
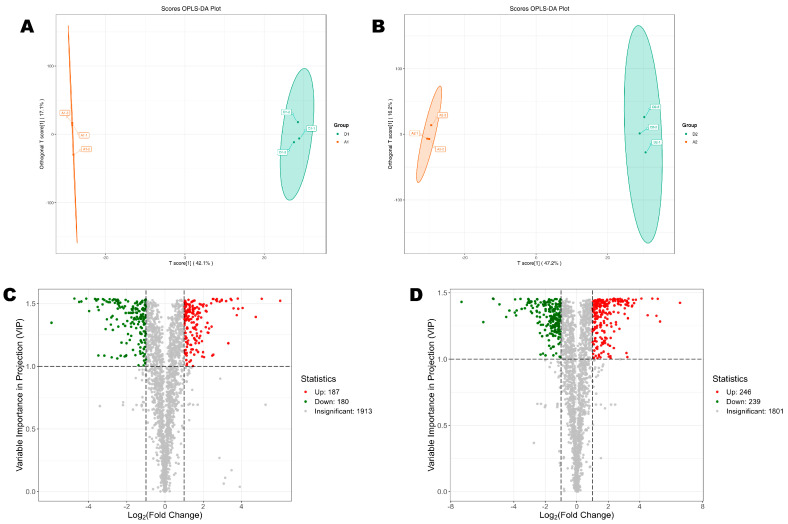
Differential metabolites (DMs). (**A**,**B**) OPLS-DA score plots of A1_vs_D1 and A2_vs_D2, respectively. (**C**,**D**) Volcano plots of DMs between A1_vs_D1 and A2_vs_D2, respectively. A1 and A2 indicate seedlings grown on the control soil on the 7th and 15th day post-germination, respectively. D1 and D2 indicate seedlings grown on the salt–alkali soil on the 7th and 15th day post-germination, respectively.

To confirm the differences between the metabolome patterns and identify DMs, we performed OPLS-DA analysis. The OPLS-DA score plots supported the significant difference between the metabolome patterns of seedlings grown on SAS and normal soil at the 7th and 15th DPG, respectively (Figure 4A,B). The goodness of prediction (Q2) values were higher than 0.94, and those of goodness of fit (R2Y) were equal to one (Figure S3A,B), confirming the larger proportion of the variance, robustness, and reliability of models. We identified 367 DMs, including 187 upregulated metabolites in seedlings on the 7th DPG (Figure 4C). On the 15th DPG, there were a total of 485 DMs, including 246 upregulated and 239 downregulated (Figure 4D).

**Figure 5:**
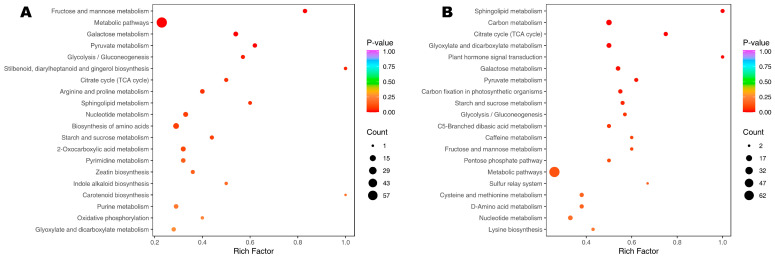
Key induced pathways in response to salt–alkali stress in wheat seedlings. KEGG enrichment result of DMs between A1_vs_D1 (**A**) and A2_vs_D2 (**B**). A1 and A2 indicate seedlings grown on the control soil on the 7th and 15th day post-germination, respectively. D1 and D2 indicate seedlings grown on the salt–alkali soil on the 7th and 15th day post-germination, respectively.

KEGG enrichment analysis helps determine the key metabolic pathways induced or altered between two different experimental conditions. The KEGG enrichment assigned the DMs on the 7th DPG mostly in biosynthesis of amino acids, nucleotide metabolism, 2-oxocarboxylic acid metabolism, pyrimidine metabolism, citrate cycle, arginine and proline metabolism, glycolysis/gluconeogenesis, galactose metabolism, starch and sucrose metabolism, sphingolipid metabolism, and pyruvate metabolism (Figure 5A). In addition to these metabolic pathways, the DMs on the 15th DPG were involved in plant hormone signal transduction, carbon metabolism, glyoxylate and dicarboxylate metabolism, carbon fixation in photosynthetic organisms, and C5-branched dibasic acid metabolism (Figure 5B). For more molecular insight, we performed KEGG analysis of the 195 common DMs. The results further highlighted the importance of the biosynthesis of secondary metabolites, carbon metabolism, biosynthesis of amino acids, nucleotide metabolism, 2-oxocarboxylic acid metabolism, biosynthesis of cofactors, citrate cycle, ascorbate and aldarate metabolism, glycolysis/gluconeogenesis, galactose metabolism, starch and sucrose metabolism, and pyruvate metabolism pathways in salt–alkali tolerance of wheat seedlings (Figure S4).

### 3.5. Potential Salt–Alkali Stress Alleviating Metabolites in Wheat

**Figure 6:**
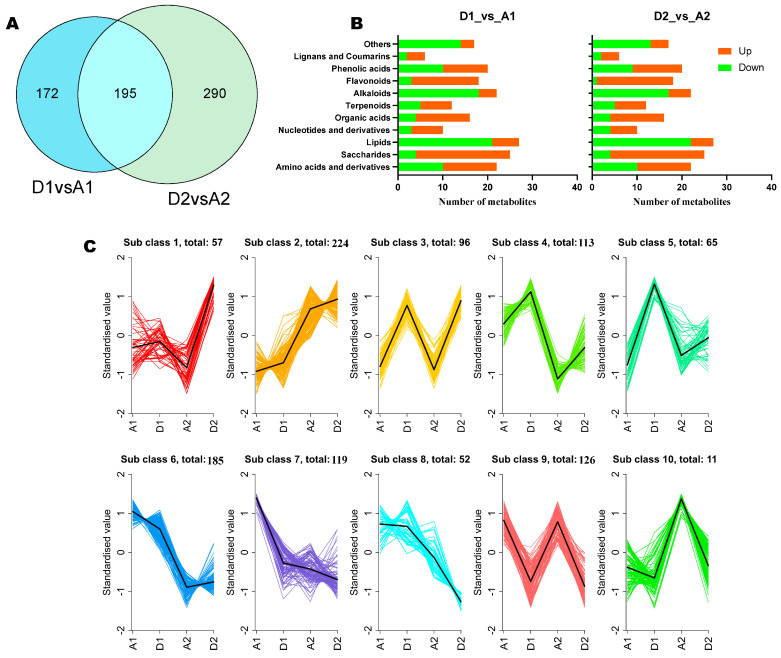
Key differential metabolites (DMs) in response to salt–alkali stress in wheat seedlings. (**A**) Venn diagram showing the common DMs in A1_vs_D1 and A2_vs_D2. (**B**) Classification of common DMs. (**C**) K-mean analysis of DMs between A1_vs_D1 and A2_vs_D2. A1 and A2 indicate seedlings grown on the control soil on the 7th and 15th day post-germination, respectively. D1 and D2 indicate seedlings grown on the salt–alkali soil on the 7th and 15th day post-germination, respectively.

To screen out potential metabolites for boosting wheat tolerance to salt–alkali stress, we searched for common DMs on the 7th and 15th DPG. The result revealed that there were 195 common differentially expressed metabolites between the 7th and 15th DPG (Figure 6A). Of them, 98 were significantly up-regulated in seedlings grown on SAS (Table S2). The classification of the common DMs disclosed that saccharides, flavonoids, phenolic acids, amino acids and derivatives, and organic acids were the main up-regulated DMs in seedlings grown on SAS (Figure 6B). Finally, we performed K-mean analysis, and the result showed that DMs classified in sub-group 3 accumulated highly in seedlings grown on SAS, which aligned with the common metabolites (Figure 6C).

**Figure 7:**
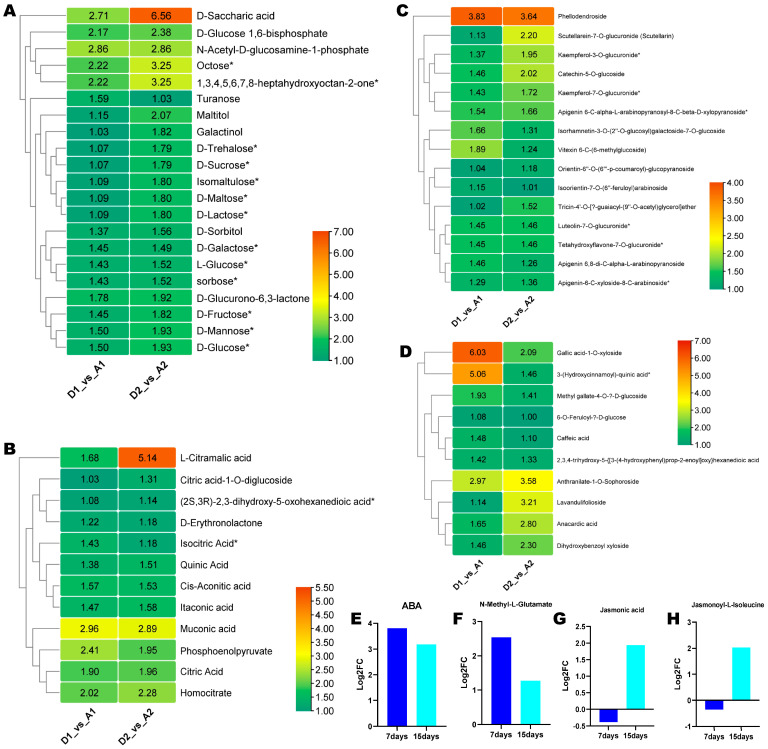
Key upregulated differential metabolites (DMs) in response to salt–alkali stress in wheat. (**A**) Saccharides. (**B**) Organic acids. (**C**) Flavonoids. (**D**) Phenolic acids. (**E**) ABA (abscisic acid). (**F**) N-methyl-L-glutamate. (**G**) Jasmonic acid. (**H**) Jasmonoyl-isoleucine. Log_2_FC values are presented. A1 and A2 indicate seedlings grown on the control soil on the 7th and 15th day post-germination, respectively. D1 and D2 indicate seedlings grown on the salt–alkali soil on the 7th and 15th day post-germination, respectively. * Does not indicate any specific thing.

Based on the above results, we focused on upregulated saccharides, flavonoids, phenolic acids, amino acids and derivatives, and organic acids in seedlings grown on SAS. The upregulated saccharides included key abiotic stress-responsive metabolites, such as saccharic acid, trehalose, sucrose, glucose, etc. (Figure 7A). Notably, saccharic acid was significantly induced by 2.71- and 6.56-fold in seedlings grown on SAS on the 7th and 15th DPG, respectively (Figure 7A). The up-regulated organic acids included mainly citrate cycle intermediates and L-citramalic acid (most induced), muconic acid, and itaconic acid (Figure 7B). The most induced flavonoids in seedlings grown on SAS included phellodendroside, scutellarein-7-*O*-glucuronide (scutellarin), catechin-5-*O*-glucoside, kaempferol-3-*O*-glucuronide, kaempferol-7-*O*-glucuronide, etc. (Figure 7C). Among the up-regulated phenolic acids, anthranilate-1-*O*-sophoroside, lavandulifolioside, anacardic acid, and dihydroxybenzoyl xyloside showed increasing accumulation patterns in seedlings grown on SAS (Figure 7D). Gallic acid-1-O-xyloside and 3-(hydroxycinnamoyl)-quinic acid were the most induced phenolic acids on the 7th DPG, with significantly reduced expression levels later (Figure 7D). Of the upregulated amino acids and derivatives, N-methyl-L-glutamate was 2.53- and 1.27-fold induced in seedlings grown on SAS on the 7th and 15th DPG, respectively (Figure 7F).

Plant hormones ABA and jasmonates are master regulators of salt stress. We found that ABA was 3.8- and 3.18-fold induced in seedlings grown on SAS on the 7th and 15th DPG, respectively (Figure 7E). JA (jasmonic acid) and JA-ILE (jasmonoyl-isoleucine) were the two identified jasmonates. Both JA and JA-ILE were down-regulated in seedlings grown on SAS on the 7th DPG (Figure 7G,H). In contrast, on the 15th day after germination, they were up-regulated by 1.93- and 2.03-fold, respectively (Figure 7G,H).

## 4. Discussion

Boosting major crop production is of utmost importance to secure food, fuel, and feed for the rapidly growing population [24,25,26]. Therefore, addressing climate change-induced challenges for sustainable agriculture has become a global priority. Accordingly, this study delved into the saline–alkaline tolerance mechanisms of wheat seedlings in arid regions using a self-developed high-generation salt–alkali wheat variety (011). This variety was chosen for the present study because it is one of the major cultivated wheat varieties in the inland arid desert regions of China, where soil salinization impairs crop productivity [4,5,6]. Despite saline–alkaline stress being more harmful to crops than salt stress alone, limited studies have been conducted on wheat to understand its response mechanisms. Soil salinization is worse in arid regions, generating saline–alkaline stress, which severely affects plant growth and crop productivity [1,2,3]. Lin found that saline–alkaline stress significantly impairs wheat seed germination and seedlings’ growth [16]. Here, the seed germination rate on SAS (87%) was slightly lower than under control conditions (97%), but the difference was not significant, which supports the tolerance potential of the variety to saline–alkaline stress. The seedling height and fresh weight on the 15th DPG under SAS were significantly lower compared to under control conditions, which is consistent with previous reports. Despite the growth reduction, seedlings appeared normal and healthy. This suggests that the growth reduction might be a tolerance mechanism of wheat seedlings to saline–alkaline stress, as more resources and energy might be mobilized to sustain molecular mechanisms of tolerance.

Physiological and metabolomics analyses revealed that wheat seedlings’ responses to prolonged saline–alkaline stress are dynamically regulated. The metabolome profile of seedlings grown on SAS on the 7th day differed from that on the 15th day, while both significantly differed from those of seedlings grown on the control soil. Previous studies revealed that plants address saline–alkaline stress by activating two key pathways: the osmotic regulation and energy metabolism [3,10,12]. Stimulation of these pathways leads to metabolic adjustment related to ion transport, photosynthesis, phytohormone synthesis, antioxidant response, solute accumulation, and osmotic adjustment. Consistent, we found that the contents of proline, soluble sugars, some organic acids, flavonoids, phenolic acids, and other amino acids and derivatives were significantly up-regulated in seedlings grown on SAS. These metabolites might be crucial for cellular homeostasis and antioxidant responses under saline–alkaline stress in wheat. It was discovered that proline and soluble sugars are vital for wheat tolerance to saline–alkaline stress [16]. Osmolytes or osmoprotectants preserve plant cell functioning and improve plant stress tolerance by balancing ionic transport, modulating enzyme activity, and scavenging ROS (reactive oxygen species) to prevent membrane disintegration [27,28]. For instance, exogenous proline application promotes salt tolerance in wheat via regulating hormonal balance, osmolytes, and antioxidant defense [29]. Flavonoids improve salt tolerance by enhancing ROS scavenging [30]. Apart from proline, we identified other potential metabolites for alleviating saline–alkaline stress in wheat, including saccharic acid, trehalose, sucrose, glucose, L-citramalic acid, muconic acid, phellodendroside, scutellarin, lavandulifolioside, N-methyl-L-glutamate, etc. Sugars are key signaling metabolites that regulate plant growth and tolerance to various biotic and abiotic stresses [31,32]. Exogenous trehalose application improves abiotic stress tolerance [31]. *Glutamate Dehydrogenase 2* has recently been identified as a hub regulator of salt–alkali and salt tolerance in cotton [33], implying that exogenous glutamate application may improve wheat salt–alkali tolerance. Further validation of these metabolites’ effects in wheat salt–alkali tolerance is required.

KEGG enrichment analysis assigned the DMs mostly to metabolic pathways related to osmotic regulation and energy metabolism. These results are in line with the above findings and previous reports in other plants [3,10,12]. Starch and sucrose metabolism, sphingolipid metabolism, and plant hormone signal transduction were among the most induced pathways. Integrated metabolome and transcriptome analysis reveals that plant hormone signaling and starch and sucrose metabolism were mostly induced in winter rapeseed (*Brassica rapa*) under high saline–alkali stress conditions [18]. Plants utilize diverse signaling components and sensors to alleviate salt stress, most of which involve Ca^2+^ sensing and signaling [14,15]. It is demonstrated that GIPC sphingolipids regulate saline–alkaline tolerance by promoting Ca^2+^ signaling [3]. Signal transduction pathways of salt–alkali stress include principally the SOS (salt overly sensitive) pathway, which regulates Ca^2+^ signaling and ion homeostasis, and ABA and protein kinase pathways, which mainly modulate osmotic readjustments [3,10]. We identified three up-regulated phytohormones (ABA, JA, and JA-ILE) in wheat seedlings grown on SAS. ABA was over 3-fold significantly induced on both the 7th and 15th DPG, while jasmonates were only induced (1.93- and 2.03-fold, respectively) on the 15th DPG. These results indicate that ABA is the master hormone regulator of salt–alkali tolerance in wheat seedlings. ABA is the most critical abiotic stress-responsive hormone [34]. ABA regulates salt–alkali tolerance by controlling the expression of several key genes involved in osmosis, ion flux, and ROS detoxification [3,10]. These results show that exogenous ABA treatment or inducing ABA synthesis and signaling may improve the salt–alkali tolerance of wheat varieties. Jasmonates are well-known phytohormones essential for plant salinity and various other stress tolerance [35]. *Streptomyces sp.* Jrh8–9 enhanced salt–alkali tolerance in alfalfa by inducing IAA and JA signaling [36]. Crosstalk between ABA and jasmonate is crucial for salt and drought tolerance in *Caragana korshinskii* [37]. These findings show that jasmonates may act as second key messengers in regulating salt–alkali tolerance in wheat seedlings. Future genomic studies are needed to identify key regulatory genes for deciphering the molecular network controlling salt–alkali tolerance in wheat.

## 5. Conclusions

This study comprehensively explored the dynamic physiological mechanisms and metabolome changes in wheat seedlings grown on SAS. We found that wheat tolerance to prolonged saline–alkaline stress at the seedling stage relies on dynamic control of growth and metabolic processes, including synthesis and accumulation of osmoprotectants (proline, soluble sugars, other amino acids, etc.), antioxidant response, synthesis of signaling and abiotic stress regulatory molecules (phytohormones, key saccharides, etc.), and synthesis and accumulation of secondary metabolites (flavonoids and phenolic acids). Notably, the up-regulation of sphingolipid metabolism, saccharides, flavonoids, organic acids (citrate cycle-related), phenolic acids, amino acids and derivatives, and stress-responsive phytohormones was essential for seedlings’ tolerance to SAS. Potential key salt–alkali stress-alleviating metabolites (saccharic acid, trehalose, sucrose, glucose, L-citramalic acid, muconic acid, phellodendroside, scutellarin, anthranilate-1-*O*-sophoroside, lavandulifolioside, N-methyl-L-glutamate, etc.) in wheat were identified. ABA is the prime regulator of salt tolerance in wheat seedlings, followed by jasmonates (JA and JA-ILE). Our results deepen the understanding of wheat tolerance mechanisms to saline–alkaline stress at the seedling stage. Moreover, they provide resources to boost wheat production on saline–alkaline lands.

## Figures and Tables

**Table 1:** Physicochemical properties of the experimental soils.

Treatment	pH	Cl^−^ mg/g	SO_4_^2−^ mg/g	Ca^2+^ mg/g	K^+^ mg/g	Mg^2+^ mg/g	Na^+^ mg/g	CO_3_^2−^ mg/g	HCO_3_^−^ mg/g	Total Salt mg/g
Control	7.21 ± 0.026	2.162 ± 0.103	2.179 ± 0.085	0.026 ± 0.003	0.021 ± 0.001	0.003 ± 0.001	4.694 ± 0.125	0.069 ± 0.015	0.07 ± 0.01	9.79 ± 0.115
SAS	10.43 ± 0.021	3.457 ± 0.096	3.428 ± 0.12	0.019 ± 0.002	0.029 ± 0.004	0.002 ± 0.001	7.459 ± 0.201	0.136 ± 0.013	0.055 ± 0.004	15.94 ± 0.137

Values are mean ± standard deviation. SAS, salt–alkali soil.

## Data Availability

All the data generated or analyzed during this study are included in this published article and its Supplementary Materials files.

## Supplementary Materials

The following supporting information can be downloaded at: https://www.mdpi.com/article/10.3390/metabo15070430/s1, Figure S1: Correlations among QC (quality control) samples; Figure S2: Classification of all identified metabolites in wheat seedlings; Figure S3: Permutation plots of OPLS-DA score plots of A1_vs_D1 (A) and A2_vs_D2 (B); Figure S4: KEGG analysis of the 195 common differential metabolites; Table S1: List of all identified metabolites in wheat seedlings; Table S2: List of the common DMs in A1_vs_D1 and A2_vs_D2.
