# Supplementary material for: Unravelling the Dynamic Physiological and Metabolome Responses of Wheat (Triticum aestivum L.) to Saline–Alkaline Stress at the Seedling Stage
Source: Metabolites. 2025 Jun 23;15(7):430. doi: 10.3390/metabo15070430 (PMC12299655; doi:10.3390/metabo15070430)
Supplement: Supplementary file 1 [file metabolites-15-00430-s001.zip › Supplementary Figures.pdf]

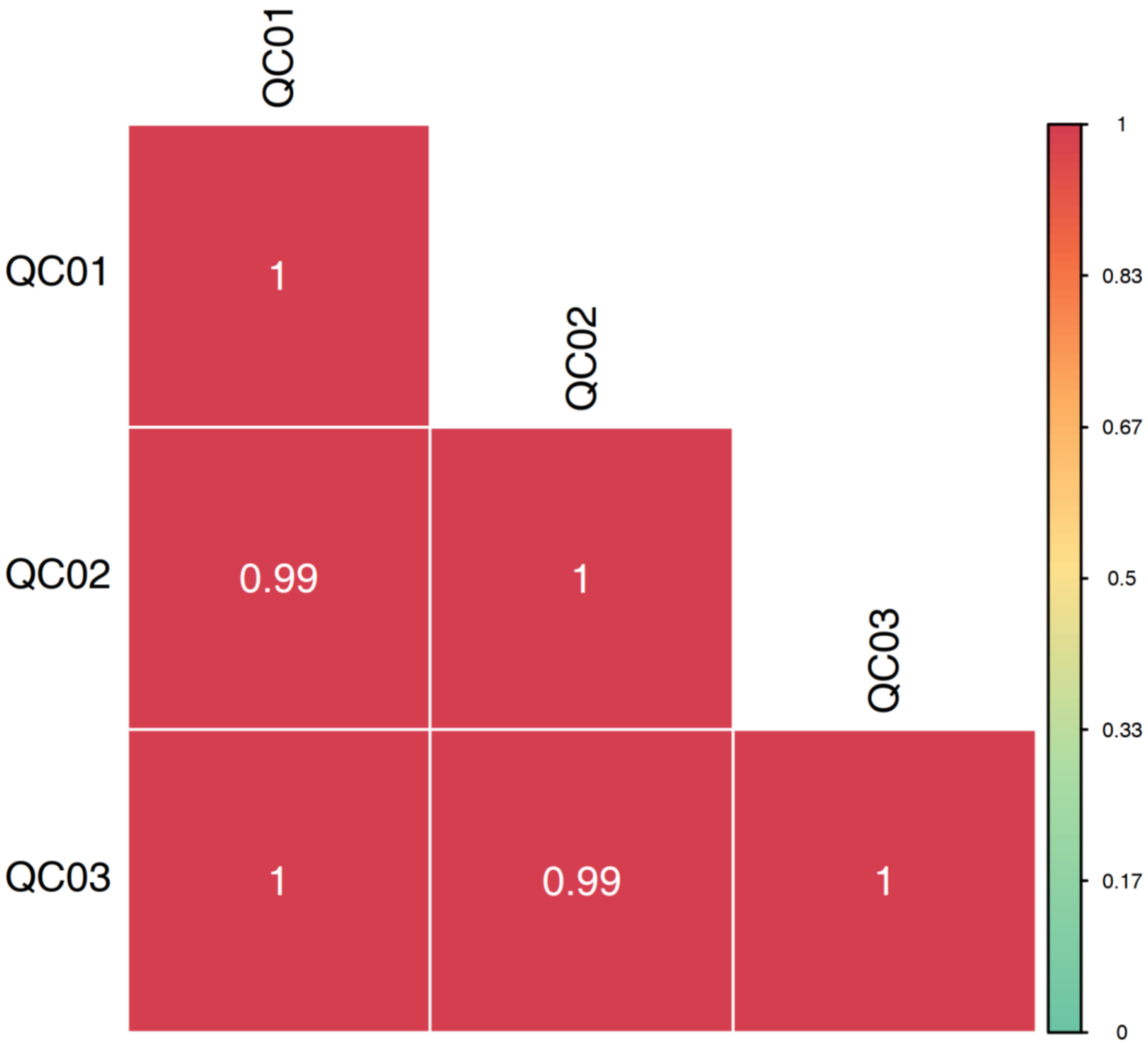

Fig. S1. Correlations among QC (quality control) samples.

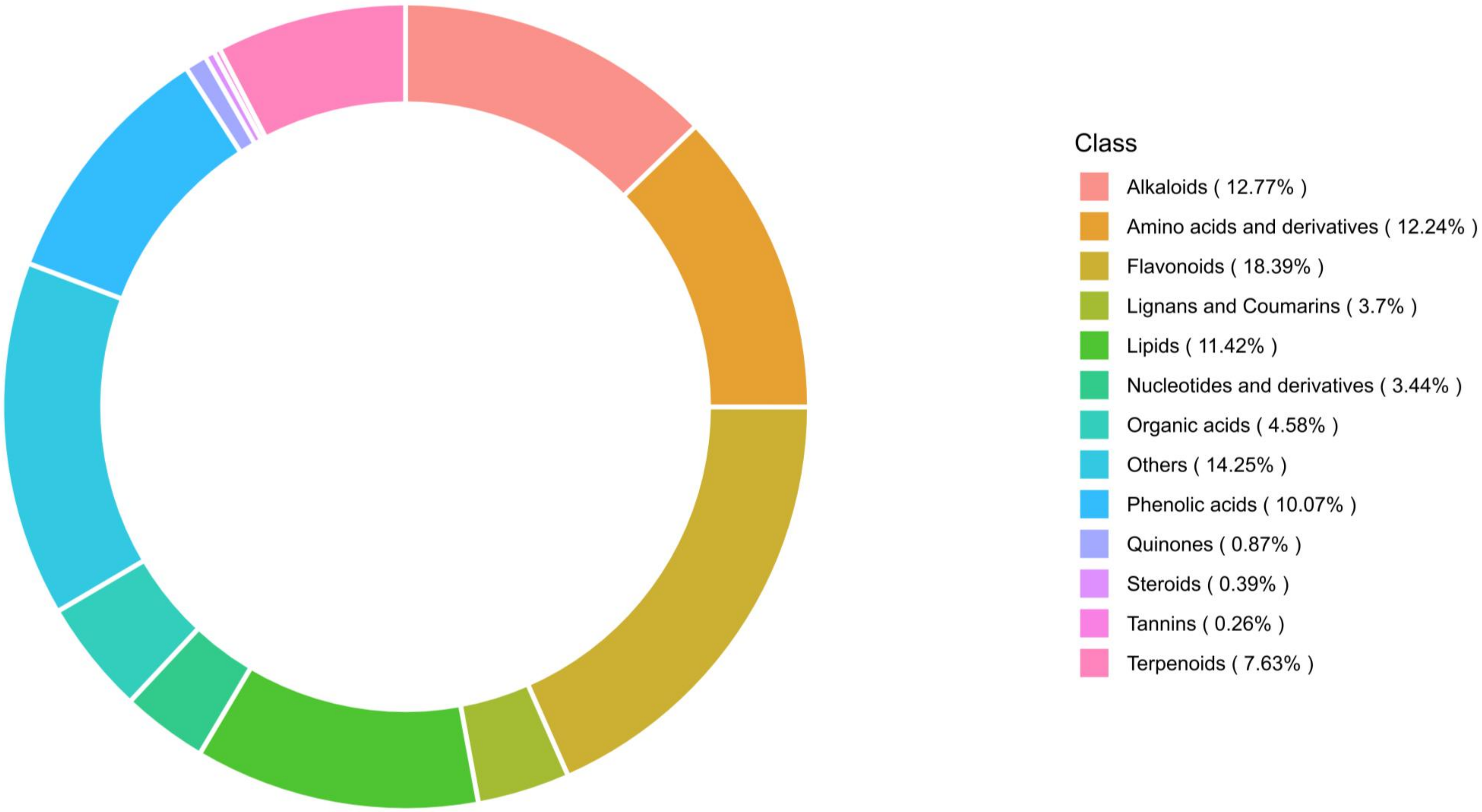

Fig. S2. Classification of all identified metabolites in wheat seedlings.

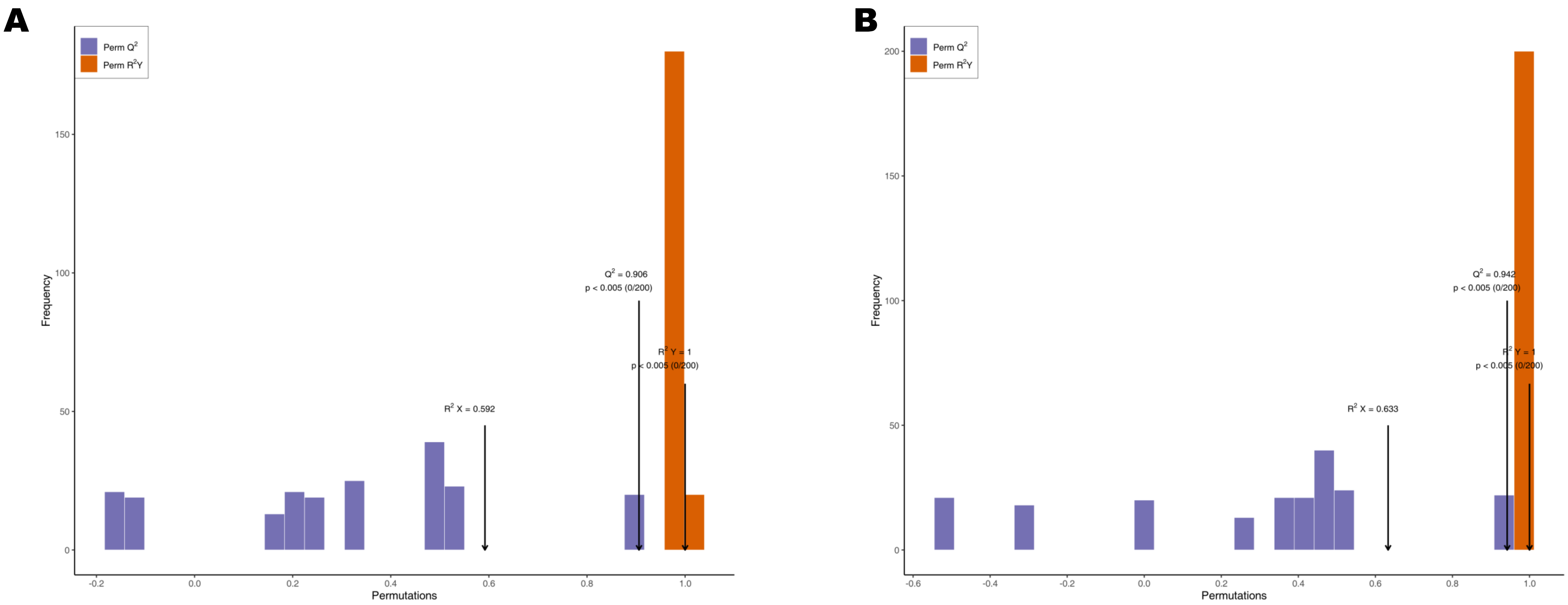

Fig. S3. Permutation plots of OPLS-DA score plots of A1_vs_D1 (A) and A2_vs_D2 (B). A1 and A2 indicate seedlings grown on the control soil on the 7th and 15th day post-germination, respectively. D1 and D2 indicate seedlings grown on the salt-alkali soil on the 7th and 15th day post-germination, respectively.

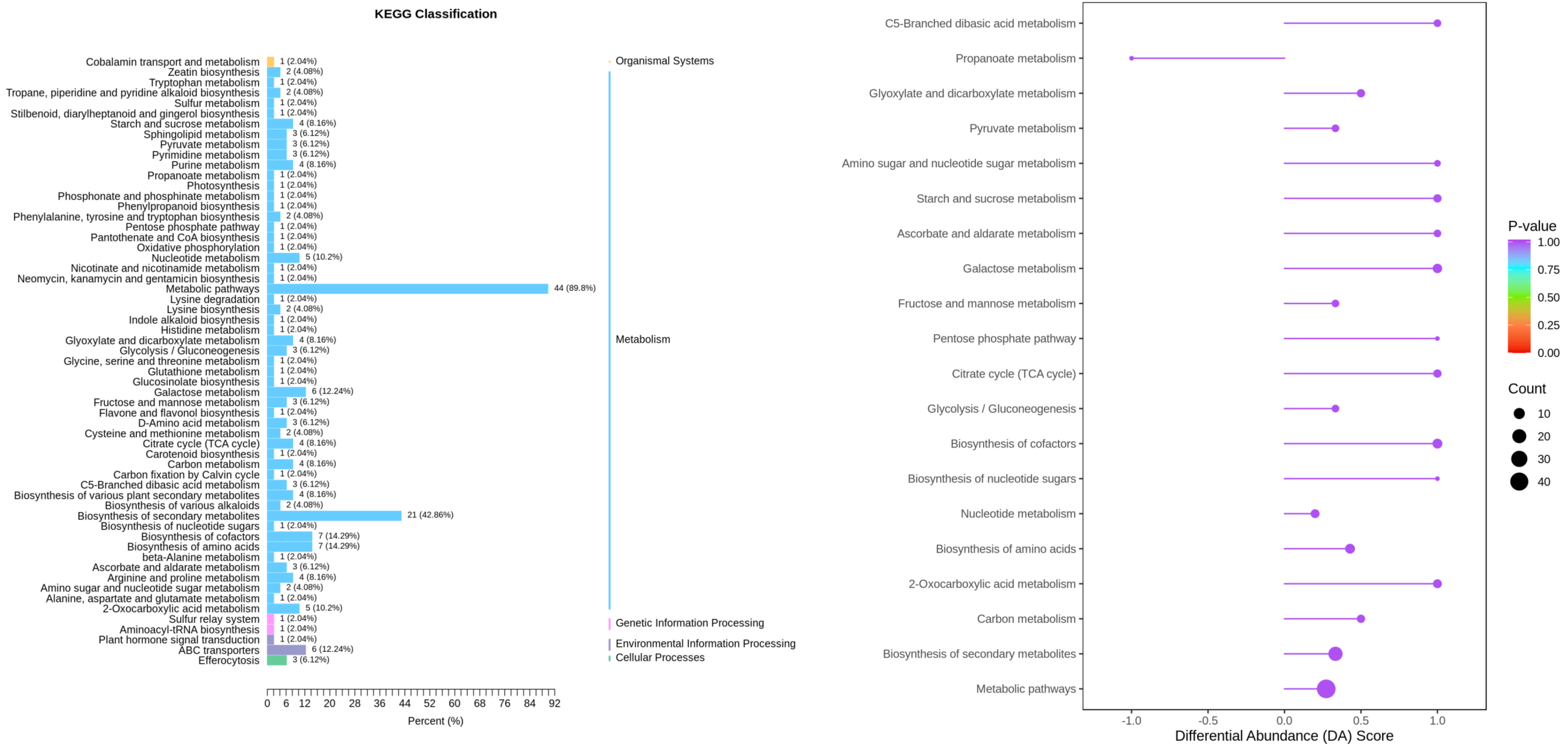

Fig. S4. KEGG analysis of the 195 common differential metabolites.
